# Experimental and Computational Evaluation of Hybrid Bi_2_O_3_/WO_3_ Nanoparticle-Filled Epoxy Composites for Lead-Free Tc-99m Gamma-Ray Shielding in Occupational Radiation Protection

**DOI:** 10.3390/polym18151804

**Published:** 2026-07-23

**Authors:** Suphalak Khamruang Marshall, Phuchisa Tepnarin, Wuttipat Wattanaphonpinich, Waritthon Atsawasetthini

**Affiliations:** Department of Radiology, Faculty of Medicine, Prince of Songkla University, Songkhla 90110, Thailand

**Keywords:** Bi_2_O_3_ nanoparticles, epoxy resin, gamma-ray attenuation, Hp(10) dosimetry, hybrid nanocomposites, lead-free radiation shielding, Phy-X/PSD, Tc-99m, WO_3_, XCOM

## Abstract

Lead-free polymer composites containing high-atomic-number fillers are promising alternatives to conventional lead shielding for nuclear medicine applications. In this study, Bi_2_O_3_-, WO_3_-, and hybrid Bi_2_O_3_/WO_3_ nanoparticle-filled epoxy resin composites were fabricated and evaluated for attenuation of the 140 keV photons emitted by technetium-99m (Tc-99m). The synthesized Bi_2_O_3_ and WO_3_ nanoparticles exhibited hydrodynamic diameters of 638.2 ± 11.3 and 404.2 ± 3.2 nm, respectively, with polydispersity indices below 0.30 and zeta potentials of −33.73 ± 0.63 and −32.47 ± 0.75 mV, indicating acceptable dispersion characteristics and colloidal stability. SEM–EDX confirmed successful incorporation of Bi- and W-containing phases into the epoxy matrix, while the XRD and FTIR analyses verified retention of the crystalline metal oxide phases and the principal chemical structure of the cured epoxy network. Tensile testing revealed a composition-dependent strength–ductility relationship, with the Bi_2_O_3_-filled composite exhibiting the highest tensile strength among the developed formulations and the hybrid composite showing the greatest elongation at break. XCOM and Phy-X/PSD simulations demonstrated that increasing high-Z filler content enhanced the mass and linear attenuation coefficients and reduced the half-value layer, tenth-value layer, and mean free path. Experimental shielding performance was evaluated using Hp(10) measurements with optically stimulated luminescence dosimeters positioned on an anthropomorphic thorax phantom under a fixed Tc-99m exposure geometry. The transmitted dose decreased with increasing filler loading, and nanoparticle-filled formulations generally outperformed the corresponding conventional-particle composites. The hybrid 75:25 Bi_2_O_3_/WO_3_ NP composite exhibited the lowest mean Hp(10) value of 0.016 µSv, corresponding to a 50% reduction relative to the lead reference under the investigated geometry. The combined structural, mechanical, computational, and dosimetric results demonstrate that hybrid filler design enables simultaneous optimization of attenuation efficiency and mechanical tolerance. These findings identify the Bi-rich hybrid epoxy composite as a promising lead-free material for customized shielding components, including vial holders, syringe-shield housings, protective panels, and workstation accessories used during Tc-99m handling in nuclear medicine.

## 1. Introduction

Epoxy resins are an important class of thermosetting polymers widely used as matrices for advanced composite materials because of their excellent adhesion, dimensional stability, chemical resistance, low curing shrinkage, and favorable mechanical performance [1]. From a polymer chemistry perspective, epoxy resins are characterized by the presence of reactive epoxide, or oxirane, functional groups in their molecular structure. These strained three-membered cyclic ether rings readily undergo ring-opening reactions with suitable curing agents, such as amines, anhydrides, phenols, or thiols, resulting in the formation of a highly cross-linked three-dimensional polymer network [2,3]. The resulting thermoset structure provides high rigidity, thermal stability, solvent resistance, and long-term dimensional integrity, which are essential properties for structural and functional composite applications. One of the most commonly used epoxy systems is based on diglycidyl ether of bisphenol A (DGEBA), which is synthesized from bisphenol A and epichlorohydrin. DGEBA-based epoxy resin contains aromatic rings, ether linkages, hydroxyl groups, and terminal epoxide groups [4]. The monomeric DGEBA structure is primarily composed of carbon, hydrogen, and oxygen, with a representative molecular formula of C_21_H_24_O_4_ and a molecular weight of approximately 340.4 g/mol, although commercial epoxy resins typically contain oligomeric species with higher molecular weights [5]. During curing with amine-based hardeners, the epoxide rings react with active hydrogen atoms in primary and secondary amine groups to form C–N bonds and hydroxyl-containing cross-linked structures [6,7]. The final properties of the cured epoxy network are governed by the molecular structure of the resin, epoxide equivalent weight, hardener chemistry, resin-to-hardener ratio, curing conditions, and crosslink density. Despite their excellent mechanical and chemical properties, neat epoxy resins are composed mainly of low-atomic-number elements, namely carbon, hydrogen, and oxygen [8]. Consequently, their intrinsic gamma-ray attenuation capability is limited. However, epoxy resin is an attractive matrix for radiation-shielding composites because it can incorporate high-density inorganic fillers while maintaining moldability, structural integrity, and dimensional stability after curing. By dispersing high-atomic-number fillers within the epoxy network, the effective atomic number and density of the resulting composite can be increased, thereby improving photon interaction probability and radiation attenuation performance [9,10].

Radiation-shielding materials are essential in medical, industrial, and nuclear medicine applications to reduce occupational and environmental exposure to ionizing radiation. In nuclear medicine, personnel may be exposed to external photon radiation during radiopharmaceutical preparation, quality control, dispensing, administration, imaging, and radioactive-waste handling. Technetium-99m (Tc-99m) is among the most widely used diagnostic radionuclides because of its favorable nuclear properties, including a short physical half-life and gamma emission suitable for nuclear imaging. However, its principal gamma emission of ~140 keV can contribute to occupational radiation exposure, particularly in high-throughput nuclear medicine departments. Therefore, optimized shielding materials are required to minimize dose to workers while maintaining practicality, safety, and ease of handling [11].

Lead and lead-containing materials have traditionally been used for photon shielding because of their high density and high atomic number [12]. Nevertheless, lead presents several limitations, including toxicity, environmental persistence, high weight, poor flexibility, limited processability, and disposal concerns [13,14,15]. These drawbacks have stimulated the development of lead-free radiation-shielding materials based on polymer composites filled with non-toxic or less hazardous high-Z compounds. Polymer-based shielding composites offer several practical advantages, including lower weight, corrosion resistance, moldability, tunable geometry, and compatibility with customized shielding designs such as syringe shields, vial holders, protective panels, wearable components, and modular shielding barriers [16,17].

Bismuth oxide (Bi_2_O_3_) and tungsten oxide (WO_3_) are promising lead-free inorganic fillers for radiation-shielding applications. Bismuth has a high atomic number and favorable photon attenuation capability, while tungsten possesses high density, high atomic number, and strong interaction probability with diagnostic-energy photons. When incorporated into an epoxy matrix, Bi_2_O_3_ and WO_3_ can enhance gamma-ray attenuation primarily through photoelectric absorption and Compton scattering [18,19]. At the diagnostic photon-energy range, including the Tc-99m energy region, the photoelectric effect is particularly important because its probability increases strongly with atomic number. Therefore, Bi_2_O_3_- and WO_3_-filled epoxy composites are expected to provide substantially better attenuation performance than neat epoxy resin [20].

Furthermore, the use of nanoparticles as fillers may provide additional advantages compared with conventional micron-sized powders. Nanoparticles have high specific surface area and can improve interfacial contact between the inorganic filler and the polymer matrix, potentially enhancing dispersion uniformity, mechanical reinforcement, and shielding homogeneity [21]. However, nanoparticle-filled composites also present processing challenges because the high surface energy of nanoparticles can promote agglomeration, particularly at high filler loading. Poor dispersion can produce voids, weak interfacial regions, reduced mechanical strength, and non-uniform radiation attenuation. Therefore, systematic characterization of particle size, polydispersity index, zeta potential, morphology, crystalline structure, elemental composition, and chemical bonding is necessary to understand the relationship between nanoparticle properties, composite structure, and shielding performance [22,23]. Moreover, hybrid filler systems have gained increasing interest for the design of multifunctional radiation-shielding composites. Compared with single-filler systems, hybrid fillers may provide improved formulation flexibility by combining the complementary properties of different high-Z materials [9,24]. In the case of Bi_2_O_3_/WO_3_-filled epoxy composites, the hybrid approach may allow optimization of radiation attenuation, density, dispersion behavior, mechanical performance, and manufacturability. Adjusting the Bi_2_O_3_:WO_3_ ratio provides a strategy to balance shielding efficiency with composite integrity, particularly for applications requiring lead-free and processable shielding materials.

In addition to experimental characterization, theoretical simulation provides valuable insight into photon attenuation behavior. Computational tools such as XCOM and Phy-X/PSD can be used to estimate shielding parameters, including mass attenuation coefficient, linear attenuation coefficient, half-value layer, tenth-value layer, mean free path, effective atomic number, effective electron density, and radiation protection efficiency [25,26]. These parameters help predict the influence of material composition, density, and photon energy on shielding performance. However, simulation alone cannot fully represent practical composite behavior because real materials may contain filler agglomerates, interfacial defects, thickness variation, voids, and geometry-dependent scattering effects. Therefore, experimental validation using dosimetric measurements is required to confirm the practical shielding performance of newly developed composites.

In this study, bismuth oxide nanoparticles (Bi_2_O_3_ NPs) and tungsten oxide nanoparticles (WO_3_ NPs) were synthesized and incorporated into epoxy resin to fabricate single-filler and hybrid Bi_2_O_3_/WO_3_ nanoparticle-filled epoxy composites for Tc-99m radiation shielding. The nanoparticles and composites were systematically characterized using dynamic light scattering (DLS), zeta potential analysis, field-emission scanning electron microscopy coupled with energy-dispersive X-ray spectroscopy (FE-SEM–EDX), X-ray diffraction (XRD), Fourier transform infrared spectroscopy (FTIR), and tensile testing. Lead-free radiation-shielding materials based on polymer composites containing high-atomic-number (high-Z) compounds have attracted increasing interest as alternatives to conventional lead-based shielding. Therefore, this study aimed to develop and comprehensively evaluate Bi_2_O_3_-, WO_3_-, and hybrid Bi_2_O_3_/WO_3_ nanoparticle-filled epoxy resin composites as lead-free shielding materials for Tc-99m gamma radiation. Radiation-shielding performance was theoretically evaluated at 140 keV using the U.S. National Institute of Standards and Technology (NIST) XCOM Photon Cross Sections Database and Physics Software/Photon Shielding and Dosimetry (Phy-X/PSD; accessed 1 March 2026). The theoretical findings were experimentally validated using optically stimulated luminescence dosimeters positioned on an anthropomorphic thorax phantom to assess the personal dose equivalent at a depth of 10 mm in human tissue [Hp(10)], representing the whole-body personal dose equivalent. By integrating polymer-composite fabrication, nanoparticle characterization, structural and chemical analysis, mechanical testing, theoretical attenuation modeling, and experimental Hp(10) dosimetry, this work provides a systematic framework for understanding the structure–property–shielding relationships of high-Z nanoparticle-filled epoxy composites for nuclear medicine radiation-protection applications.

## 2. Materials and Methods

### 2.1. Materials

Bismuth nitrate pentahydrate [Bi(NO_3_)_3_·5H_2_O, 98.0%] was purchased from KemAus, Cherrybrook, NSW, Australia. Sodium hydroxide (NaOH), oxalic acid (C_2_H_2_O_4_) and sodium sulfate (Na_2_SO_4_) were obtained from Thermo Fisher Scientific, Waltham, MA, USA. Bismuth oxide (Bi_2_O_3_, 99.5%, analytical reagent grade) and oxalic acid (99.5%, analytical reagent grade) were supplied by Q RëC™, QRëc Chemical Co., Ltd., Auckland, New Zealand. Tungsten oxide (WO_3_) was purchased from Thermo Fisher Scientific, Waltham, MA, USA. Sodium tungstate dihydrate (Na_2_WO_4_·2H_2_O) was purchased from Carlo Erba Reagents, Cornaredo, Milan, Italy. Hydrochloric acid (HCl, 37%) was obtained from RCI Labscan Limited, Pathumwan, Bangkok, Thailand. Deionized water used throughout the experiments was prepared using a Direct-Q 3 water purification system. All solvents used in this study were purchased from MilliporeSigma, St. Louis, MO, USA, and Thermo Fisher Scientific, Waltham, MA, USA.

### 2.2. Preparation of Hybrid Bi_2_O_3_/WO_3_ Nanoparticle-Filled Epoxy Resin Composites

Bi_2_O_3_ NPs were synthesized using a precipitation method. Briefly, Bi(NO_3_)_3_·5H_2_O and Na_2_SO_4_ were dissolved in 500 mL of deionized water and stirred magnetically for 2 h. Separately, NaOH was dissolved in 500 mL of deionized water to prepare the alkaline precipitating solution. The NaOH solution was then added dropwise to the bismuth-containing solution at a controlled and constant rate under continuous stirring until complete precipitation occurred. The resulting precipitate was collected by filtration using filter paper, washed with 70% ethanol, and oven-dried at 150 °C for 2 h to obtain dry Bi_2_O_3_ nanoparticle powder.

WO_3_ NPs were prepared using an acid-precipitation method. A 3.0 M hydrochloric acid (HCl) solution was prepared by diluting concentrated HCl (37%) with deionized water. Separately, Na_2_WO_4_ was dissolved in 500 mL of deionized water under continuous stirring until a clear solution was obtained. Subsequently, 100 mL of 3.0 M HCl was added dropwise to the sodium tungstate solution under continuous stirring until a yellow precipitate formed. After precipitation, oxalic acid (C_2_H_2_O_4_) was added, and the mixture was stirred for 1 h. Oxalic acid was used as a complexing and morphology-regulating agent to coordinate tungsten species, control nucleation and particle growth, and support the formation of WO_3_ nanoparticles during the acid-precipitation process. The reaction vessel was then covered with aluminum foil and maintained at 95 °C for 12 h. The resulting precipitate was collected by filtration and oven-dried at 60 °C for 4 h to obtain dry WO_3_ nanoparticle powder.

**Figure 1:**
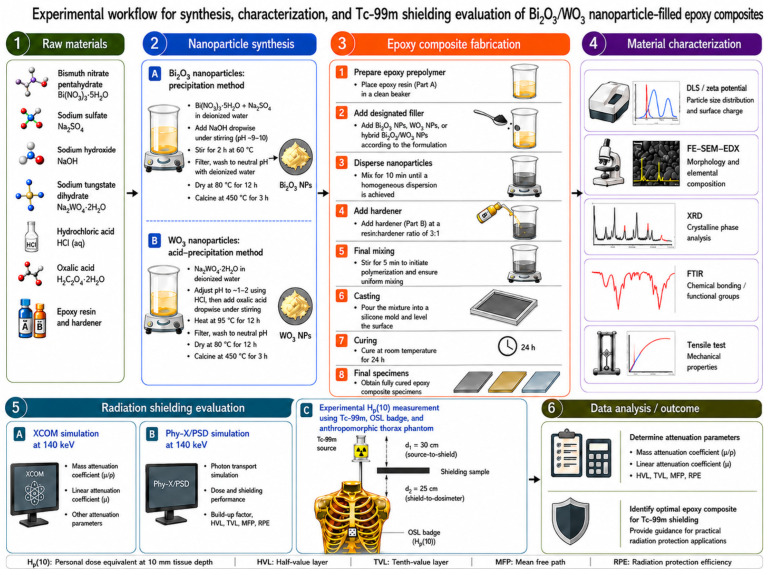
Experimental workflow for the synthesis, characterization, and Tc-99m radiation-shielding evaluation of Bi_2_O_3_/WO_3_ nanoparticle-filled epoxy composites. The workflow summarizes the major experimental stages, including raw material preparation, synthesis of Bi_2_O_3_ and WO_3_ nanoparticles, fabrication of epoxy composites containing single and hybrid nanoparticle fillers, physicochemical and mechanical characterization, radiation-shielding simulation using XCOM and Phy-X/PSD at 140 keV, experimental Hp(10) measurement using Tc-99m, an OSL badge, and an anthropomorphic thorax phantom, and final data analysis to determine attenuation parameters and identify the optimal shielding formulation. The experimental geometry used a source-to-shield distance (d_1_) of 30 cm and a shield-to-dosimeter distance (d_2_) of 25 cm.

Epoxy resin composites containing Bi_2_O_3_ NPs, WO_3_ NPs, and hybrid Bi_2_O_3_/WO_3_ NPs were fabricated using a conventional mixing and curing method. The epoxy prepolymer was first prepared, after which the designated filler, either Bi_2_O_3_ NPs, WO_3_ NPs, or hybrid Bi_2_O_3_/WO_3_ NPs, was added and mixed for 10 min until a homogeneous dispersion was achieved (Table 1). For the hybrid Bi_2_O_3_/WO_3_ formulations, the synthesized Bi_2_O_3_ NPs and WO_3_ NPs were first weighed according to the designed Bi_2_O_3_ ratios of 25:75, 50:50, and 75:25, while maintaining the total filler loading at 100 parts per hundred parts of epoxy resin (phr). The weighed Bi_2_O_3_ and WO_3_ nanoparticle powders were then premixed to obtain a uniform hybrid filler blend before being gradually incorporated into the epoxy prepolymer. Polymerization was initiated by adding the hardener, followed by additional stirring for 5 min. The resulting mixture was then poured into molds and cured at room temperature for 24 h to obtain fully cured epoxy composite specimens (Figure 1).

### 2.3. Particle Size and Zeta Potential Analysis of Bi_2_O_3_ and WO_3_ Nanoparticles

The particle size distribution and zeta potential of Bi_2_O_3_ NPs and WO_3_ NPs were analyzed using DLS and electrophoretic light scattering, respectively. Briefly, each nanoparticle sample was dispersed in deionized water at a concentration of 0.5 mg/mL. The suspensions were homogenized using a bath sonicator (Branson Ultrasonics^TM^ CPX Series Ultrasonic Cleaning Bath, Model CPX1800, Branson Ultrasonics Corporation, Brookfield, CT, USA) operating at 40 kHz with a maximum ultrasonic power of 70 W for 15 min at room temperature. During sonication, the sample tubes were kept fully immersed in the sonication bath to ensure uniform ultrasonic energy transfer and to promote homogeneous nanoparticle dispersion. After sonication, the samples were filtered through a 0.2 µm polyvinylidene fluoride (PVDF) membrane filter to remove large aggregates and impurities. Following sample preparation, 1 mL of each suspension was transferred into a disposable cuvette or zeta cell for measurement. The measurement temperature was maintained at 25 °C. Particle size and zeta potential analyses were performed using a ZetaPALS analyzer (Brookhaven Instruments Corporation, Nashua, NH, USA). For particle size analysis, measurements were conducted at a scattering angle of 173°. The refractive index and viscosity of the dispersant were set to 1.330 and 0.890 cP, respectively.

### 2.4. Morphological and Elemental Characterization by FE-SEM–EDX

The surface morphology and elemental composition of the neat epoxy, Bi_2_O_3_, Bi_2_O_3_ NPs, WO_3_, and WO_3_ NPs were examined using FE-SEM–EDX. SEM imaging was performed using an FE-SEM Apreo system (FEI Technologies Inc., Hillsboro, OR, USA), while elemental analysis was conducted using an EDX detector from Oxford Instruments NanoAnalysis, High Wycombe, UK.

Prior to analysis, the samples were carefully cleaned to remove surface contaminants. The neat epoxy, Bi_2_O_3_ NPs, WO_3_ NPs and hybrid Bi_2_O_3_/WO_3_ NPs were rinsed with ethanol and then sonicated for 10–15 min to remove loosely attached impurities and improve sample cleanliness. After sonication, the samples were washed with deionized water and dried in a vacuum desiccator to remove residual moisture. Complete drying was necessary to minimize charging effects and prevent image distortion during SEM observation. The dried samples were mounted onto SEM stubs and sputter-coated with a thin gold layer of ~20 nm to improve electrical conductivity. After coating, the samples were placed in a vacuum desiccator for 15 min to remove any remaining moisture before SEM analysis. The prepared samples were then introduced into the SEM chamber, and high-resolution micrographs were acquired to evaluate their surface morphology, particle features, and structural characteristics. Elemental composition was further investigated using EDX analysis. Particular attention was given to the detection of bismuth and oxygen in Bi_2_O_3_ NPs, tungsten and oxygen in WO_3_ NPs, and the major elemental components of the neat epoxy. The EDX spectra were used to confirm the presence and distribution of the expected elements in the prepared materials.

### 2.5. Structural and Crystalline Phase Characterization by XRD

For XRD analysis, neat epoxy resin and hybrid Bi_2_O_3_/WO_3_ NPs were prepared as solid powder samples. Each material was finely ground using an agate mortar and pestle to obtain a homogeneous powder with particle sizes below 10 µm, thereby reducing preferred orientation effects during diffraction analysis. The powdered samples were then pressed into flat, uniform pellets using a hydraulic press at 5 tons for 2 min. Care was taken to ensure that each pellet had a smooth and even surface to improve diffraction quality. The prepared pellets were placed into the XRD sample holder, and diffraction patterns were recorded using Cu-Kα radiation with a wavelength of λ = 1.5406 Å. The samples were scanned over a 2θ range of 10–90°. The crystalline phases of the samples were identified by comparing the observed diffraction peak positions and relative intensities with standard crystallographic databases.

### 2.6. Chemical Bonding and Molecular Structure Analysis by FTIR

FTIR was performed to investigate the molecular structure and chemical bonding characteristics of the prepared samples. FTIR spectra were acquired using a VERTEX 70 spectrometer (Bruker Daltonics GmbH & Co. KG, Bremen, Germany) equipped with an attenuated total reflectance (ATR) accessory. Spectral data were collected over the wavenumber range of 4000–400 cm^−1^, with 64 scans recorded for each measurement. All measurements were performed using a deuterated L-alanine-doped triglycine sulfate (DLaTGS) detector with potassium bromide (KBr) windows. The FTIR spectra were analyzed to identify characteristic functional groups and chemical bonds present in the neat epoxy, nanoparticle fillers, and composite samples. Particular attention was given to the appearance, disappearance, or shifting of absorption bands, which may indicate chemical interactions, structural changes, or bonding modifications within the composite materials.

### 2.7. Mechanical Characterization of Composite Materials by Tensile Testing

Tensile testing was conducted to determine the mechanical performance of the lead, neat epoxy resin and epoxy composite specimens containing Bi2O3 NPs, WO3 NPs, and hybrid Bi2O3/WO3 NPs. Dumbbell-shaped specimens were prepared and trimmed according to the ISO 37 Type II standard [27]. Testing was performed using a universal testing machine (ZwickRoell Z010, Zwick GmbH & Co. KG, Ulm, Germany) equipped with a 1 kN load cell. A preload of 0.1 N was applied before measurement, and the specimens were tested at a crosshead speed of 100 mm/min with a gauge length of 20 mm. Ultimate tensile strength and elongation at break were calculated from the stress–strain data.

### 2.8. Radiation Shielding Simulation and Measurements

#### 2.8.1. XCOM-Based Simulation of Materials for Tc-99m Radiation Shielding

Theoretical radiation-shielding parameters of the PbO_2_, neat epoxy resin and epoxy composites containing Bi_2_O_3_, WO_3_, and hybrid Bi_2_O_3_/WO_3_ were calculated using the XCOM photon cross-section database provided by the National Institute of Standards and Technology (NIST) [25]. XCOM allows the calculation of photon cross sections and attenuation coefficients for elements, compounds, and mixtures over photon energies from 1 keV to 100 GeV. For each formulation, the elemental or compound composition was entered into XCOM based on the relative mass fractions of PbO_2_, neat epoxy, Bi_2_O_3_, and WO_3_ in the composite system. For hybrid composites, the Bi_2_O_3_:WO_3_ filler ratios were entered according to the designed formulation, including 25:75, 50:50, and 75:25 ratios.

#### 2.8.2. Phy-X/PSD-Based Simulation of Materials for Tc-99m Radiation Shielding

Radiation shielding parameters of the neat epoxy resin and epoxy composites containing Bi_2_O_3_, WO_3_, and hybrid Bi_2_O_3_/WO_3_ were further evaluated using the Phy-X/PSD online software (accessed 1 March 2026) [26]. Phy-X/PSD is a photon shielding and dosimetry platform developed for calculating shielding parameters, including the mass attenuation coefficient (MAC), linear attenuation coefficient (LAC), half-value layer (HVL), tenth-value layer (TVL), mean free path (MFP), effective electron density (Neff), atomic cross-section (ACS) and electronic cross-section (ECS) over a wide photon-energy range.

For each sample formulation, the elemental composition and corresponding weight fractions were entered into the Phy-X/PSD platform. The compositions were defined based on the epoxy matrix and the incorporated filler system, including Bi_2_O_3_, WO_3_, or hybrid Bi_2_O_3_/WO_3_. For the hybrid composites, the Bi_2_O_3_:WO_3_ filler ratios were set according to the prepared formulations, namely 25:75, 50:50, and 75:25. The measured density of each cured composite specimen was also entered into the software to allow calculation of density-dependent shielding parameters. Additionally, the simulated results obtained from Phy-X/PSD were compared among the neat epoxy resin, single-filler Bi_2_O_3_ and WO_3_ composites, and hybrid Bi_2_O_3_/WO_3_ composites to determine the effect of filler type, filler loading, and hybrid filler ratio on gamma-ray attenuation performance.

#### 2.8.3. Experimental Analysis of Materials for Tc-99m Radiation Shielding

**Figure 2:**
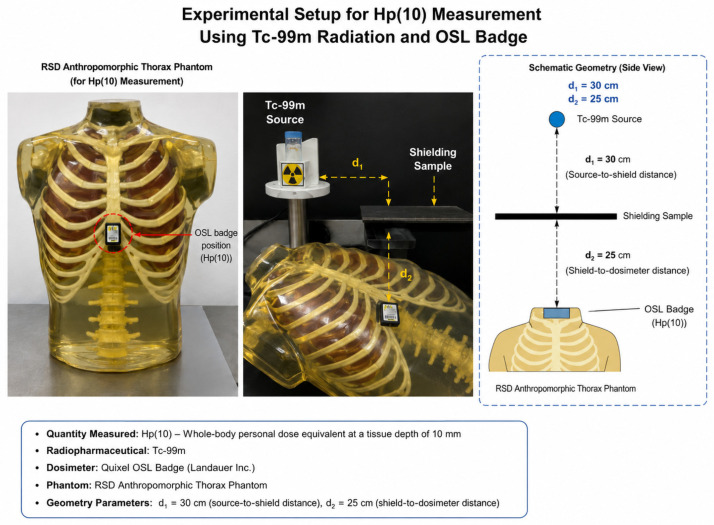
Experimental setup for Hp(10) radiation-shielding measurement using Tc-99m and an OSL badge. An RSD anthropomorphic thorax phantom was used to simulate whole-body occupational exposure conditions for nuclear medicine personnel. The OSL badge was positioned on the thoracic region of the phantom to assess Hp(10), corresponding to the whole-body personal dose equivalent at a tissue depth of 10 mm. The shielding sample was placed between the Tc-99m source and the dosimeter, with a source-to-shield distance (d_1_) of 30 cm and a shield-to-dosimeter distance (d_2_) of 25 cm. The geometry was maintained consistently for all shielding measurements.

The experimental setup consisted of Quixel optically stimulated luminescence (OSL) badges (Landauer Inc., Glenwood, IL, USA) and Tc-99m radiopharmaceuticals. Anthropomorphic phantoms were used to simulate the anatomical characteristics of nuclear medicine personnel during radiation exposure. An RSD anthropomorphic thorax phantom was used to assess Hp(10), corresponding to the whole-body personal dose equivalent at a tissue depth of 10 mm (Figure 2).

### 2.9. Statistical Analysis

Statistical analyses were performed using GraphPad Prism version 10.0 (GraphPad Software Inc., Boston, MA, USA). Each experiment was independently repeated three to ten times, depending on the experimental endpoint, and the results are expressed as mean ± standard deviation (SD). For two-group comparisons, statistical significance was evaluated using Student’s *t*-test. For comparisons among multiple groups or multiple experimental factors, one-way or two-way analysis of variance (ANOVA) was used, followed by the Student–Newman–Keuls post hoc test. Differences were considered statistically significant at *p* ≤ 0.05. Significance levels are indicated as ns for *p* > 0.05 and * for *p* ≤ 0.05.

## 3. Results

### 3.1. Structural and Morphological Characterization of Hybrid Bi_2_O_3_/WO_3_ Nanoparticle-Filled Epoxy Resin Composites

#### 3.1.1. Particle Size Distribution and Zeta Potential of Bi_2_O_3_ and WO_3_ Nanoparticles

The synthesized Bi_2_O_3_ NPs and WO_3_ NPs were analyzed by DLS and zeta potential measurements to evaluate their dispersion characteristics prior to incorporation into the epoxy matrix. These parameters are critical for nanoparticle-filled polymer composites because particle size, distribution uniformity, and surface charge can affect filler dispersion, interfacial compatibility, mechanical behavior, and radiation-shielding performance.

**Figure 3:**
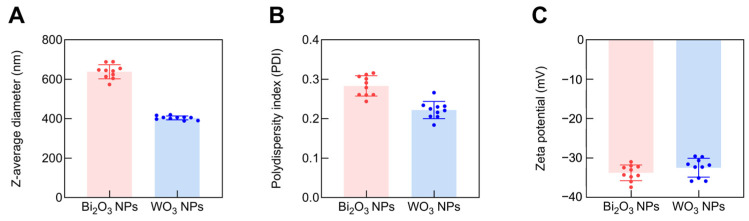
Particle size distribution and colloidal stability of synthesized Bi_2_O_3_ and WO_3_ NPs. (**A**) Z-average hydrodynamic diameter, (**B**) polydispersity index (PDI), and (**C**) zeta potential of Bi_2_O_3_ NPs and WO_3_ NPs measured by dynamic light scattering and electrophoretic light scattering. Data are presented as mean ± SD (*n* = 10).

As shown in Figure 3A, the z-average hydrodynamic diameter of Bi_2_O_3_ NPs was 638.2 ± 11.3 nm, while WO_3_ NPs exhibited a smaller z-average diameter of 404.2 ± 3.2 nm. The intensity-weighted DLS size-distribution profiles showed a single dominant particle-size population for both Bi_2_O_3_ NPs and WO_3_ NPs, indicating an approximately unimodal distribution under the applied dispersion and measurement conditions. The larger hydrodynamic size of Bi_2_O_3_ NPs may indicate partial particle agglomeration in aqueous suspension, which is commonly observed in metal oxide nanoparticles because of their high surface energy. Nevertheless, both nanoparticle systems remained within the submicron range, supporting their applicability as functional fillers in epoxy-based shielding composites. The PDI values were 0.283 ± 0.008 for Bi_2_O_3_ NPs and 0.218 ± 0.015 for WO_3_ NPs, as presented in Figure 3B. These values indicate moderately narrow particle-size distributions, with WO_3_ NPs demonstrating greater dispersion uniformity than Bi_2_O_3_ NPs. The PDI values below 0.3 suggest acceptable dispersion quality for subsequent composite fabrication [28]. Zeta-potential analysis further confirmed the colloidal stability of the nanoparticle suspensions. As shown in Figure 3C, Bi_2_O_3_ NPs and WO_3_ NPs exhibited mean zeta-potential values of −33.73 ± 0.63 mV and −32.47 ± 0.75 mV, respectively. These values represent the mean ± standard deviation obtained from repeated measurements. The instrument-derived standard deviations of the zeta-potential distributions were 1.95 mV for Bi_2_O_3_ NPs and 1.74 mV for WO_3_ NPs, indicating relatively narrow surface-charge distributions. The absolute zeta-potential values exceeding ±30 mV indicate sufficient electrostatic repulsion between particles, suggesting moderate colloidal stability and a reduced tendency toward rapid aggregation [29,30].

Collectively, the DLS and zeta-potential results demonstrate that both Bi_2_O_3_ NPs and WO_3_ NPs possessed suitable dispersion characteristics for epoxy composite fabrication. WO_3_ NPs showed a smaller hydrodynamic diameter and lower PDI, indicating comparatively better dispersion uniformity, whereas Bi_2_O_3_ NPs maintained adequate colloidal stability despite their larger apparent particle size. These findings support the use of both nanoparticle systems as high-Z fillers for Tc-99m radiation-shielding epoxy composites.

#### 3.1.2. FE-SEM–EDX Analysis of Bi_2_O_3_/WO_3_ Nanoparticle-Filled Epoxy Resin Composites

**Figure 4:**
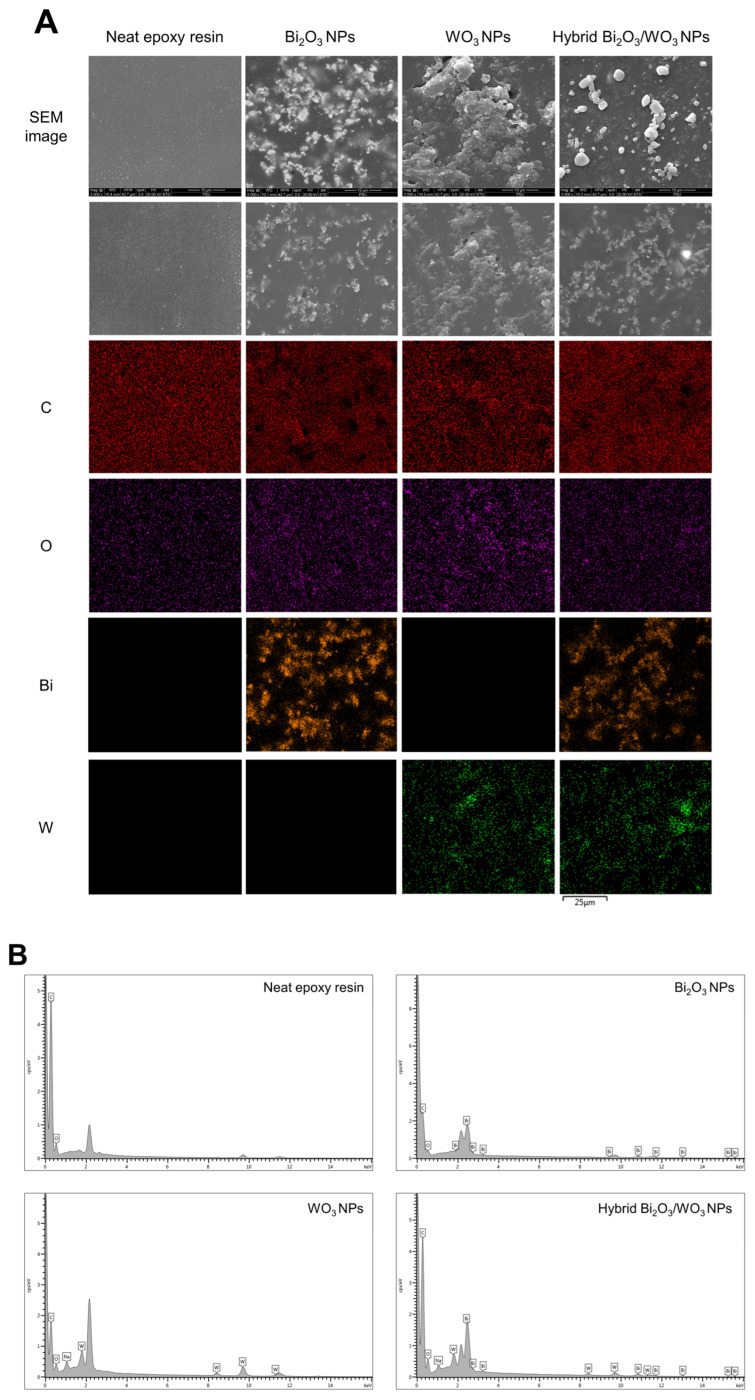
SEM–EDX characterization of neat epoxy resin and Bi_2_O_3_/WO_3_ nanoparticle-filled epoxy resin composites. (**A**) SEM images and elemental mapping of carbon (C), oxygen (O), bismuth (Bi), and tungsten (W) in neat epoxy resin, Bi_2_O_3_-filled epoxy, WO_3_-filled epoxy, and hybrid Bi_2_O_3_/WO_3_-filled epoxy composites. (**B**) Representative EDX spectra confirming the elemental composition of each sample.

FE-SEM–EDX analysis was conducted to evaluate the morphology and elemental composition of the neat epoxy resin and nanoparticle-filled epoxy composites. As shown in Figure 4A, the neat epoxy resin exhibited a continuous polymeric surface, consistent with the formation of a cured epoxy matrix. After filler incorporation, the Bi_2_O_3_- and WO_3_-filled composites showed distinguishable inorganic domains within the epoxy matrix, indicating successful loading of the respective metal oxide nanoparticles. The hybrid Bi_2_O_3_/WO_3_ composite exhibited morphological features associated with both filler systems, confirming the formation of a hybrid organic–inorganic composite structure. Additionally, the elemental mapping further supported the successful incorporation of the fillers. Carbon was distributed throughout the samples, corresponding to the epoxy matrix, while oxygen was associated with both the epoxy network and the oxide fillers. The Bi_2_O_3_-filled composite showed clear Bi signals, whereas the WO_3_-filled composite exhibited W signals. In the hybrid composite, both Bi and W were detected, verifying the simultaneous incorporation of Bi_2_O_3_ and WO_3_ nanoparticles within the epoxy matrix.

The EDX spectra in Figure 4B were consistent with the elemental mapping results. The neat epoxy resin primarily showed carbon and oxygen peaks, while the nanoparticle-filled composites exhibited characteristic Bi and/or W peaks depending on the filler composition. These elemental signatures confirm that the designed Bi_2_O_3_, WO_3_, and hybrid Bi_2_O_3_/WO_3_ filler systems were successfully incorporated into the epoxy resin.

Taken together, the FE-SEM–EDX findings provide morphological and compositional evidence for the successful fabrication of Bi_2_O_3_-, WO_3_-, and hybrid Bi_2_O_3_/WO_3_ nanoparticle-filled epoxy resin composites. The presence of high-Z Bi and W elements within the epoxy matrix supports the intended function of these composites as lead-free shielding materials for Tc-99m gamma radiation.

#### 3.1.3. XRD Characterization: Structural Insights into Bi_2_O_3_/WO_3_ Nanoparticle-Filled Epoxy Resin Composites

**Figure 5:**
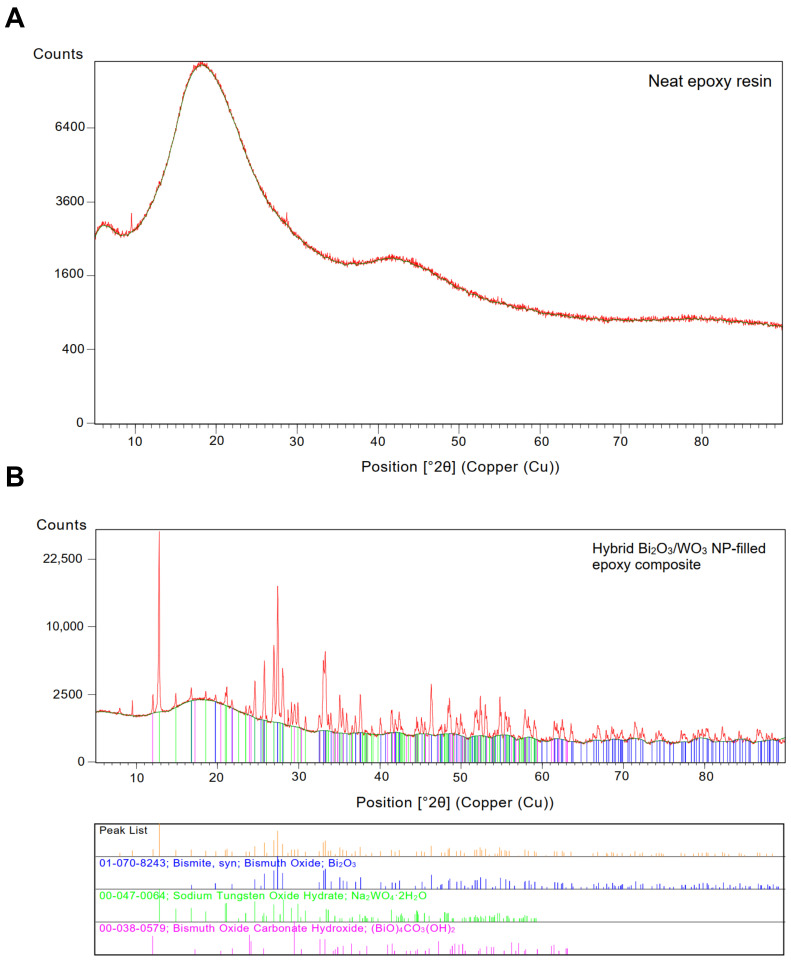
XRD patterns of neat epoxy resin and hybrid Bi_2_O_3_/WO_3_ NP-filled epoxy resin composite. (**A**) Neat epoxy resin showing a broad amorphous halo characteristic of the cured polymer matrix. (**B**) Hybrid Bi_2_O_3_/WO_3_ NP-filled epoxy composite showing distinct crystalline diffraction peaks, confirming the incorporation and retention of crystalline Bi_2_O_3_ and WO_3_ NP fillers within the epoxy matrix.

XRD analysis was performed to investigate the structural characteristics, crystalline phase formation, and filler incorporation in the neat epoxy resin and hybrid Bi_2_O_3_/WO_3_ nanoparticle-filled epoxy resin composite. As shown in Figure 5A, the neat epoxy resin exhibited a broad diffraction halo centered at 18–20° 2θ, with no sharp crystalline reflections. This pattern is typical of an amorphous polymer network and confirms the non-crystalline nature of the cured epoxy matrix. In contrast, the hybrid Bi_2_O_3_/WO_3_ NP-filled epoxy composite showed multiple sharp diffraction peaks distributed across the scanned 2θ range, as shown in Figure 5B. The appearance of intense and well-defined peaks indicates the successful incorporation of crystalline inorganic NP fillers into the epoxy matrix. The crystalline reflections were mainly associated with Bi_2_O_3_-related phases and WO_3_/tungstate-related phases, as confirmed by comparison with standard reference diffraction patterns. The presence of these peaks demonstrates that the high-atomic-number oxide fillers retained their crystalline structure after composite fabrication and curing. The XRD pattern of the hybrid composite also showed that the amorphous background of the epoxy resin remained present, although it was partially masked by the strong crystalline reflections from the incorporated NPs. This coexistence of an amorphous polymer halo and crystalline oxide peaks confirms the formation of a hybrid composite system consisting of an epoxy matrix reinforced with Bi_2_O_3_ and WO_3_-based NP fillers. Importantly, no major disappearance of the epoxy amorphous feature was observed, suggesting that the addition of the NPs did not induce crystallization of the epoxy resin or substantially alter its amorphous network structure.

The strong diffraction intensity observed in the hybrid composite reflects the high crystallinity and high loading of the inorganic fillers [31]. Since Bi and W are high-atomic-number elements, their crystalline oxide phases are expected to contribute significantly to photon attenuation through photoelectric absorption at the Tc-99m gamma energy range. Therefore, the XRD results provide structural evidence supporting the use of Bi_2_O_3_/WO_3_ NP-filled epoxy composites as potential lead-free radiation-shielding materials. Overall, the XRD findings confirm that the neat epoxy resin is predominantly amorphous, whereas the hybrid Bi_2_O_3_/WO_3_-filled epoxy composite contains distinct crystalline phases corresponding to the incorporated metal oxide NPs. These results support the successful fabrication of a hybrid amorphous–crystalline composite structure, in which the epoxy resin provides the polymer matrix and the crystalline Bi_2_O_3_/WO_3_ fillers provide radiation-attenuating functionality.

#### 3.1.4. FTIR-Based Chemical Bonding Analysis of Bi_2_O_3_/WO_3_ Nanoparticle-Filled Epoxy Resin Composites

**Figure 6:**
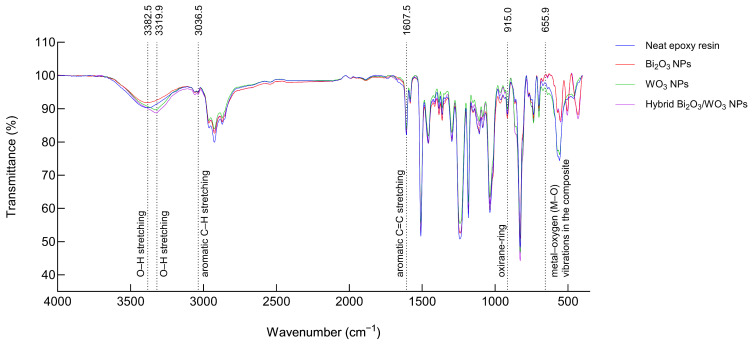
FTIR spectra of neat epoxy resin and Bi_2_O_3_-, WO_3_-, and hybrid Bi_2_O_3_/WO_3_ NP-filled epoxy composites.

FTIR spectroscopy was used to evaluate the chemical bonding characteristics of neat epoxy resin and epoxy composites containing Bi_2_O_3_ NPs, WO_3_ NPs, and hybrid Bi_2_O_3_/WO_3_ fillers. The spectra were recorded in the 4000–400 cm^−1^ wavenumber range and are presented as transmittance (%) versus wavenumber (cm^−1^). The reported band positions therefore correspond to FTIR wavenumber values rather than transmittance-scale values. As shown in Figure 6, all samples retained the characteristic absorption bands of the epoxy matrix, indicating preservation of the principal polymer structure after nanoparticle incorporation. The broad bands at approximately 3382.5–3319.9 cm^−1^ were assigned to O–H stretching vibrations arising from hydroxyl groups, adsorbed moisture, and surface hydroxyl species on the metal oxide nanoparticles. The bands near 3036.5 and 1607.5 cm^−1^ corresponded to aromatic C–H and C=C stretching, respectively, confirming retention of the aromatic epoxy backbone. The band at approximately 915.0 cm^−1^ was attributed to oxirane-ring vibrations and may reflect residual epoxy functionality within the cured network.

In the low-wavenumber region, the nanoparticle-filled composites exhibited a distinct absorption band near 655.9 cm^−1^. This feature was assigned to overlapping metal–oxygen lattice vibrations, including W–O–W modes, typically observed within ~600–800 cm^−1^, and Bi–O or Bi–O–Bi modes, generally located within the 400–650 cm^−1^ region. Its increased prominence in the single-filler and hybrid composites confirmed the incorporation of Bi_2_O_3_ and WO_3_ into the epoxy matrix. Because these vibrational regions overlap, the band is most appropriately described as a combined metal–oxygen vibration rather than being attributed exclusively to either oxide phase. No major peak disappearance or substantial spectral shifts were observed, indicating that nanoparticle incorporation did not significantly alter the principal chemical structure of the cured epoxy network. Overall, the FTIR results support the successful formation and chemical compatibility of the Bi_2_O_3_-, WO_3_-, and hybrid Bi_2_O_3_/WO_3_-filled epoxy composites.

Taken together, the FTIR data demonstrate that Bi_2_O_3_ and WO_3_ nanoparticle incorporation preserved the characteristic epoxy network while introducing identifiable metal–oxygen vibrational features, confirming successful filler integration without major chemical disruption of the cured matrix.

#### 3.1.5. Tensile Testing of Bi_2_O_3_/WO_3_ Nanoparticle-Filled Epoxy Resin Composites

**Figure 7:**
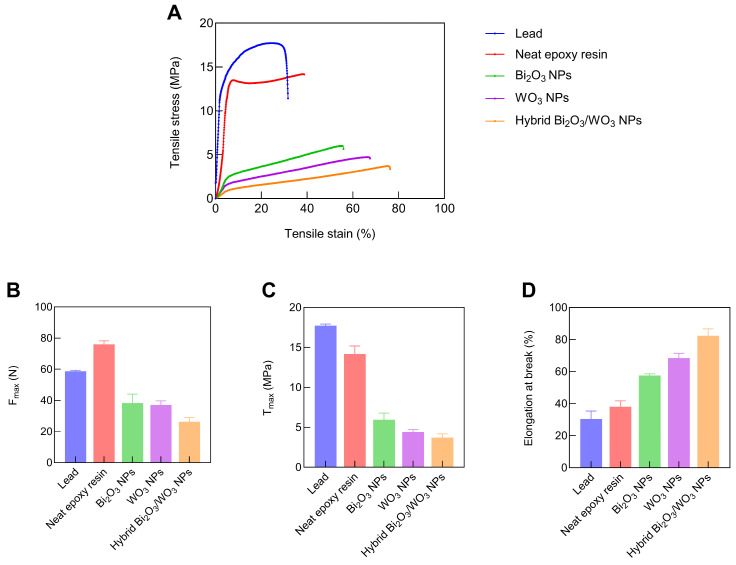
Tensile properties of lead reference material and Bi_2_O_3_/WO_3_ nanoparticle-filled epoxy resin composites. (**A**) Representative tensile stress–strain curves; (**B**) maximum force (Fmax); (**C**) maximum tensile stress (Tmax); and (**D**) elongation at break of lead, neat epoxy resin, Bi_2_O_3_ nanoparticle-filled epoxy, WO_3_ nanoparticle-filled epoxy, and hybrid Bi_2_O_3_/WO_3_ nanoparticle-filled epoxy composites. Data are presented as mean ± SD (*n* = 5).

Tensile testing was performed to evaluate the mechanical performance of the prepared nanoparticle-filled epoxy composites and to compare their behavior with that of the lead reference material. As shown in Figure 7A, the lead reference exhibited the highest tensile stress, reaching ~17–18 MPa, followed by stress reduction at failure. In contrast, the Bi_2_O_3_ NP-, WO_3_ NP-, and hybrid Bi_2_O_3_/WO_3_ NP-filled epoxy composites demonstrated lower tensile stress values but indicated more gradual deformation behavior. Among the nanoparticle-filled composites, the Bi_2_O_3_ NP-filled epoxy composite confirmed the highest tensile stress, whereas the hybrid Bi_2_O_3_/WO_3_ NP-filled epoxy composite showed the lowest tensile stress but the greatest strain tolerance.

The maximum force values shown in Figure 7B followed the order lead > Bi_2_O_3_ NP-filled epoxy ≈ WO_3_ NP-filled epoxy > hybrid Bi_2_O_3_/WO_3_ NP-filled epoxy. The lead reference exhibited the highest Fmax, 59 N, whereas the Bi_2_O_3_ NP- and WO_3_ NP-filled epoxy composites showed comparable values of 38 N and 37 N, respectively. The hybrid Bi_2_O_3_/WO_3_ NP-filled epoxy composite presented a lower Fmax, 27 N. A similar trend was observed for Tmax in Figure 7C, where the lead reference demonstrated the highest tensile strength, while the nanoparticle-filled epoxy composites showed reduced tensile strength. Among the composites, the Bi_2_O_3_ NP-filled epoxy composite exhibited the highest tensile strength, followed by the WO_3_ NP-filled and hybrid Bi_2_O_3_/WO_3_ NP-filled epoxy composites. The reduction in tensile strength after nanoparticle incorporation could be attributed to the relatively high loading of inorganic fillers, which can restrict polymer-chain mobility, disrupt the continuity of the load-bearing epoxy matrix, and promote localized stress concentration at the filler–matrix interfaces. In addition, partial agglomeration of high-density metal oxide nanoparticles may generate microstructural heterogeneity, thereby reducing effective stress transfer and weakening tensile performance. This effect can be more pronounced in the hybrid Bi_2_O_3_/WO_3_ system because differences in particle size, surface chemistry, and filler distribution can increase interfacial complexity and contribute to reduced tensile strength.

In contrast, elongation at break showed an opposite trend. As presented in Figure 7D, the lead reference revealed the lowest elongation at break 30%, whereas all nanoparticle-filled epoxy composites showed higher elongation values. The Bi_2_O_3_ NP-filled epoxy composite showed an elongation at break of 57%, the WO_3_ NP-filled epoxy composite 68%, and the hybrid Bi_2_O_3_/WO_3_ NP-filled epoxy composite 85%. The increased elongation of the epoxy-based composites suggests greater deformation capacity than the lead reference under tensile loading. The hybrid composite exhibited the highest ductility, indicating enhanced strain accommodation despite its lower tensile strength.

Therefore, the tensile results demonstrate a clear trade-off between tensile strength and deformability. The lead reference exhibited superior tensile strength but limited elongation, whereas the nanoparticle-filled epoxy composites showed lower tensile strength but improved elongation at break. Among the epoxy composites, the Bi_2_O_3_ NP-filled formulation provided the highest tensile strength, while the hybrid Bi_2_O_3_/WO_3_ NP-filled formulation presented the greatest ductility. Although the hybrid Bi_2_O_3_/WO_3_ nanoparticle-filled epoxy composite showed lower tensile strength, its higher elongation at break indicates improved deformation capacity and reduced brittleness. This behavior may be beneficial for lead-free shielding devices that require handling durability and moderate formability, such as customized syringe shields, vial holders, movable shielding panels, radiopharmaceutical transport covers, and molded shielding accessories for nuclear medicine workstations. These results suggest that hybrid filler systems may be useful where a balance between radiation attenuation, mechanical tolerance, and practical manufacturability is required.

### 3.2. Radiation Shielding Properties of Hybrid Bi_2_O_3_/WO_3_ Nanoparticle-Filled Epoxy Resin Composites

#### 3.2.1. Radiation Shielding Effectiveness Evaluation Using the XCOM Computational Model

**Figure 8:**
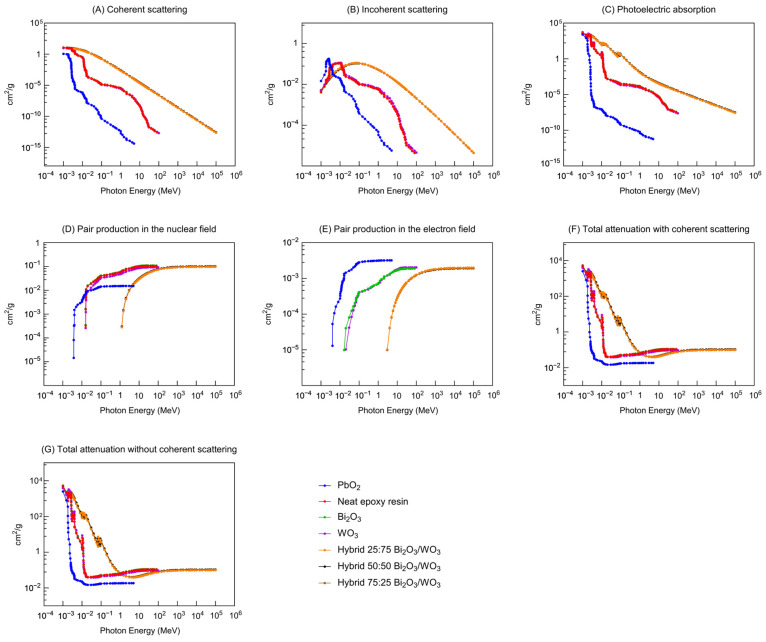
XCOM-calculated photon-interaction and attenuation parameters of neat epoxy, PbO_2_, Bi_2_O_3_, WO_3_, and hybrid Bi_2_O_3_/WO_3_ formulations as functions of photon energy. (**A**) Coherent scattering; (**B**) incoherent scattering; (**C**) photoelectric absorption; (**D**) pair production in the nuclear field; (**E**) pair production in the electron field; (**F**) total mass attenuation coefficient including coherent scattering; and (**G**) total mass attenuation coefficient without coherent scattering.

The XCOM analysis demonstrated that photon attenuation was strongly dependent on photon energy and material composition (Figure 8). At low photon energies, photoelectric absorption was the dominant interaction mechanism, with PbO_2_ and the Bi_2_O_3_- and WO_3_-containing formulations exhibiting substantially higher MAC than neat epoxy. This behavior is attributed to the high atomic numbers of Pb, Bi, and W, which markedly increase the probability of photoelectric interaction. At the principal Tc-99m photon energy of 140 keV, the Bi_2_O_3_-, WO_3_-, and hybrid Bi_2_O_3_/WO_3_-containing formulations showed greater calculated attenuation than neat epoxy. PbO_2_ generally exhibited the highest attenuation, while the hybrid formulations produced closely grouped values, indicating that changes in the Bi ratio caused only moderate variation in mass attenuation while preserving the shielding contribution of both high-Z fillers. Therefore, coherent scattering contributed mainly at low energies, whereas incoherent scattering became more important in the intermediate-energy region. Pair production contributed only above its threshold energy and was therefore not relevant to Tc-99m photons. The total MAC consequently decreased with increasing energy as the photoelectric contribution diminished, before increasing gradually at high energies because of pair-production processes.

Overall, the XCOM results confirm that the shielding enhancement of the developed epoxy composites originates predominantly from increased photoelectric interaction at the Tc-99m energy.

#### 3.2.2. Computational Assessment of Radiation-Shielding Performance Using Phy-X/PSD

**Figure 9:**
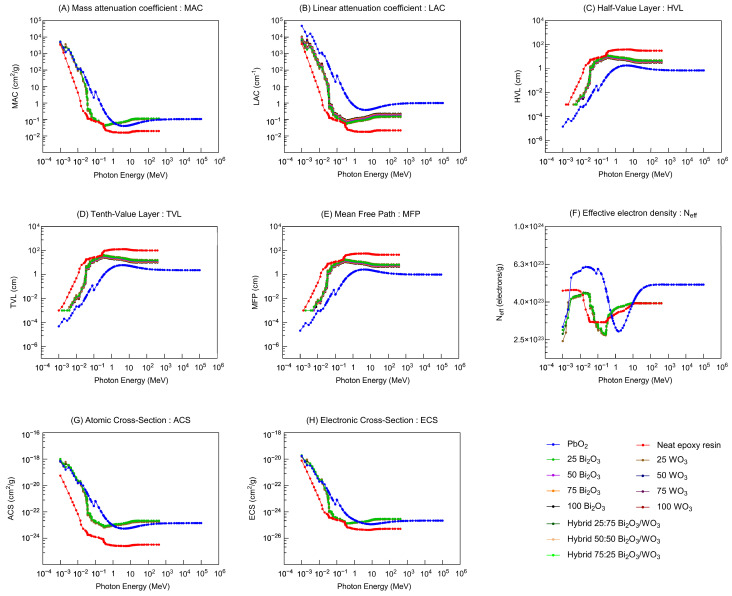
Phy-X/PSD-calculated photon-shielding parameters of neat epoxy resin, PbO_2_, Bi_2_O_3_-filled epoxy, WO_3_-filled epoxy, and hybrid Bi_2_O_3_/WO_3_-filled epoxy composites as functions of photon energy. (**A**) Mass attenuation coefficient (MAC); (**B**) linear attenuation coefficient (LAC); (**C**) half-value layer (HVL); (**D**) tenth-value layer (TVL); (**E**) mean free path (MFP); (**F**) effective electron density (Neff); (**G**) atomic cross-section (ACS); and (**H**) electronic cross-section (ECS).

Phy-X/PSD calculations demonstrated a strong dependence of photon attenuation on energy, filler composition, and filler loading (Figure 9). MAC and LAC decreased sharply with increasing photon energy in the low-energy region, reflecting the declining contribution of photoelectric absorption. Distinct variations associated with the absorption edges of Bi, W, and Pb were also evident. Neat epoxy resin consistently exhibited the lowest attenuation coefficients, whereas incorporation of Bi_2_O_3_ or WO_3_ increased both MAC and LAC. The enhancement became more pronounced at higher filler loadings, confirming that the high atomic numbers and densities of Bi- and W-containing phases increased the probability of photon interaction. At the principal Tc-99m photon energy of 140 keV, all nanoparticle-filled epoxy formulations provided greater attenuation than neat epoxy. Additionally, the PbO_2_ reference retained the highest calculated shielding performance; however, the Bi_2_O_3_-, WO_3_-, and hybrid Bi_2_O_3_/WO_3_-filled composites showed substantial improvement without the use of lead. The hybrid formulations exhibited composition-dependent behavior, indicating that their attenuation performance can be adjusted by varying the relative proportions of Bi_2_O_3_ and WO_3_.

HVL, TVL, and MFP exhibited trends inverse to those of the LAC. Neat epoxy resin showed the highest HVL, TVL, and MFP values, indicating a greater thickness requirement for effective attenuation. Increasing the metal oxide content reduced these parameters, demonstrating that nanoparticle incorporation decreased the shielding thickness required to achieve equivalent photon attenuation. Neff, ACS, and ECS varied with photon energy and elemental composition, further confirming the influence of high-Z fillers on photon interaction mechanisms. Their profiles reflected the transition from photoelectric absorption at low energies to Compton scattering at intermediate energies and pair production at higher energies.

Therefore, the Phy-X/PSD results confirm that Bi_2_O_3_ and WO_3_ incorporation significantly improves the Tc-99m shielding performance of epoxy resin. Higher filler loading increased attenuation coefficients and reduced the required shielding thickness, while the hybrid system provided a tunable lead-free formulation strategy for balancing attenuation efficiency with the mechanical and processing requirements of polymer-based radiation-shielding materials.

#### 3.2.3. Advanced Hybrid Bi_2_O_3_/WO_3_ Nanoparticle-Filled Epoxy Resin Composites for Effective Tc-99m Radiation Shielding

**Figure 10:**
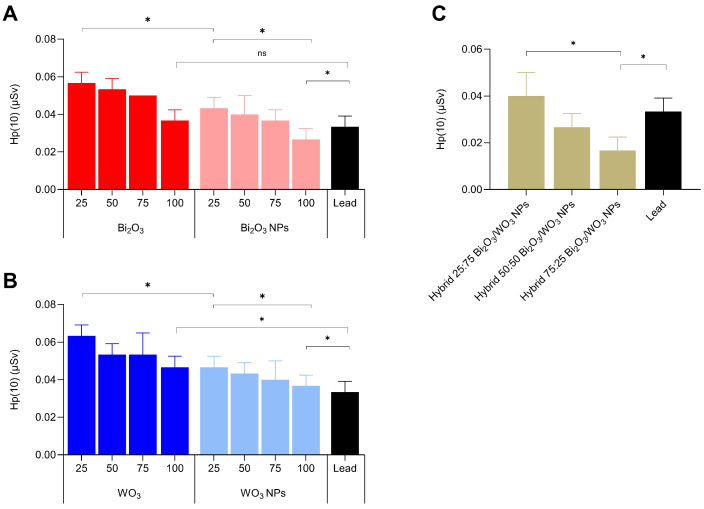
Experimental Tc-99m shielding performance of Bi_2_O_3_-, WO_3_-, and hybrid Bi_2_O_3_/WO_3_-filled epoxy resin composites based on Hp(10) measurements. (**A**) Hp(10) values for conventional Bi_2_O_3_- and Bi_2_O_3_ nanoparticle-filled epoxy composites at nominal filler loadings of 25–100%; (**B**) corresponding values for conventional WO_3_- and WO_3_ nanoparticle-filled epoxy composites; and (**C**) Hp(10) values for hybrid Bi_2_O_3_/WO_3_ nanoparticle-filled composites with Bi_2_O_3_:WO_3_ ratios of 25:75, 50:50, and 75:25 compared with the lead reference. Data are presented as mean ± SD (*n* = 3), with individual symbols representing replicate measurements. * *p* ≤ 0.05; ns, not significant (*p* > 0.05).

The experimental Tc-99m shielding performance of the epoxy composites was assessed using Hp(10), where lower values indicate reduced transmitted dose and greater attenuation efficiency. As shown in Figure 10A,B, Hp(10) decreased progressively with increasing Bi_2_O_3_ or WO_3_ loading. For both filler systems, the nanoparticle-filled composites consistently produced lower Hp(10) values than the corresponding conventional particle formulations, demonstrating improved shielding effectiveness at equivalent nominal loadings.

Bi_2_O_3_-containing composites generally exhibited stronger attenuation than the corresponding WO_3_ formulations. At 100% nominal loading, the Bi_2_O_3_ nanoparticle-filled composite produced an Hp(10) value below that of the lead reference, whereas the WO_3_ nanoparticle-filled composite remained slightly higher. These findings indicate that bismuth-rich formulations were more effective for attenuating the 140 keV photons emitted by Tc-99m under the present experimental conditions.

The hybrid composites showed a clear composition-dependent response (Figure 10C). Hp(10) decreased as the Bi_2_O_3_ proportion increased, with the hybrid 75:25 Bi_2_O_3_/WO_3_ NP formulation producing the lowest transmitted dose among all tested materials. This formulation also outperformed the lead reference, indicating that an optimized Bi_2_O_3_: WO_3_ ratio can provide highly effective lead-free shielding. Therefore, the results demonstrate that filler loading, particle scale, and hybrid composition are key determinants of Tc-99m attenuation, with the Bi-rich hybrid composite representing the most promising formulation for nuclear medicine shielding applications.

## 4. Discussion

The present study demonstrates that Bi_2_O_3_ and WO_3_ NPs can be incorporated into an epoxy thermoset matrix to produce lead-free composites with tunable physicochemical, mechanical, and Tc-99m radiation-shielding properties. The combined DLS, zeta-potential, FE-SEM–EDX, XRD, FTIR, tensile, computational, and experimental dosimetry results establish a consistent structure–property–shielding relationship governed principally by filler type, particle scale, loading level, and the relative Bi_2_O_3_/WO_3_ composition.

The synthesized Bi_2_O_3_ and WO_3_ particles exhibited z-average hydrodynamic diameters of 638.2 ± 11.3 and 404.2 ± 3.2 nm, respectively, with PDI values below 0.30 and negative zeta potentials of approximately −33 mV. These results indicate submicron dispersed particles with acceptable distribution uniformity and electrostatic stability under the aqueous measurement conditions. Additionally, the lower diameter and PDI of WO_3_ suggest comparatively greater uniformity in aqueous dispersion, whereas both oxide systems exhibited sufficient surface charge to resist rapid aggregation before composite fabrication. These nanoparticle characteristics are directly relevant to the final composite properties because particle size, PDI, and colloidal stability affect how uniformly the high-atomic-number fillers can be distributed within the epoxy matrix. A narrower particle-size distribution and adequate surface charge can reduce severe agglomeration during premixing, improve filler dispersion, and promote more homogeneous spatial distribution of Bi- and W-containing phases in the cured composite. For radiation protection, the intrinsic attenuation capability is mainly determined by the high atomic numbers and densities of Bi_2_O_3_ and WO_3_, the filler loading, sample thickness, and photon energy. However, uniform filler distribution is also important because it minimizes resin-rich regions, voids, and agglomeration-related defects that could create local attenuation heterogeneity or reduce the effective shielding path. Therefore, the acceptable DLS and zeta-potential results support the formation of structurally uniform shielding composites, which contributes to consistent Tc-99m photon attenuation and reliable Hp(10) reduction. Similar studies have emphasized that particle size and dispersion state can influence the homogeneity, mechanical integrity, and attenuation consistency of polymer shielding composites [32,33,34,35,36].

FE-SEM–EDX confirmed the presence of the intended oxide fillers within the epoxy matrix. Carbon and oxygen signals were associated with the organic matrix and oxide phases, while the characteristic Bi and W signals verified the incorporation of Bi_2_O_3_ and WO_3_, respectively. Simultaneous detection of Bi and W in the hybrid formulation confirmed successful dual-filler fabrication. Comparable FE-SEM–EDX investigations of Bi_2_O_3_-, WO_3_-, MgO-, ZnO-, SnO-, and tungsten carbide-filled epoxy systems have similarly demonstrated that elemental mapping is essential for establishing the distribution of high-Z phases and relating composite microstructure to attenuation behavior [37,38,39,40]. Although the present composites were successfully fabricated using conventional mixing, nanoparticle agglomeration remains an important consideration at high filler loading, particularly at 100 phr. Agglomerated Bi_2_O_3_ or WO_3_ particles may generate local filler-rich regions, resin-rich regions, voids, and stress-concentration sites, which can reduce mechanical integrity and produce non-uniform attenuation pathways. Therefore, the DLS, PDI, zeta-potential, and FE-SEM–EDX results are important because they provide complementary information on the initial dispersion behavior of the nanoparticles and their distribution within the cured epoxy matrix. In future optimization, agglomeration could be further reduced by gradual filler addition, high-shear mixing, planetary or vacuum-assisted mixing, three-roll milling, nanoparticle surface modification, and controlled degassing before curing. These approaches are expected to improve filler dispersion, minimize void formation, and enhance both shielding uniformity and mechanical reliability of high-loading Bi_2_O_3_/WO_3_ epoxy composites.

The structural analyses further supported composite formation. Neat epoxy resin displayed the broad diffraction halo expected for an amorphous crosslinked thermoset, whereas the filled samples retained the crystalline reflections of Bi_2_O_3_ and WO_3_. The coexistence of an amorphous epoxy background and crystalline metal oxide peaks indicates that curing did not eliminate the crystalline structures responsible for high-Z photon interactions. Moreover, FTIR spectra retained the principal aromatic, ether, hydroxyl, and epoxy-network-related bands of the cured resin, while low-wavenumber metal–oxygen vibrations supported the presence of Bi–O and W–O structures. The absence of extensive loss of the characteristic polymer bands indicates that filler incorporation did not cause major degradation of the epoxy network. Similar preservation of the polymer framework alongside identifiable inorganic phases has been reported for epoxy composites containing Bi_2_O_3_, WO_3_, MgO, ZnO, zirconium oxide, boron oxide, calcium carbonate, and tungsten carbide [10,36,38,40].

Furthermore, the tensile results revealed a strength–deformability balance associated with filler composition. The lead reference exhibited the highest maximum tensile stress, whereas the Bi_2_O_3_-filled epoxy showed the highest tensile strength among the developed composites. The hybrid Bi_2_O_3_/WO_3_ formulation exhibited lower tensile strength but the greatest elongation at break, indicating enhanced strain accommodation and reduced brittleness. High inorganic loading commonly alters polymer-chain mobility and stress transfer by replacing part of the continuous load-bearing polymer phase with rigid particles. Depending on filler concentration, particle size, interfacial adhesion, and curing conditions, this may increase hardness or stiffness while reducing tensile strength, or produce an optimum at an intermediate loading [41,42,43,44]. The comparatively high elongation of the hybrid composite suggests that combining the two oxide phases modified stress redistribution within the epoxy matrix. Additionally, curing conditions are also important for the long-term performance of epoxy-based shielding composites. In the present study, all composite specimens were cured at room temperature for 24 h to provide a practical fabrication route for customized radiation-protection components. Although this curing condition enabled comparison among the different filler systems under identical preparation conditions, controlled thermal post-curing may further increase the crosslink density of the epoxy network and improve mechanical strength, thermal stability, dimensional stability, and long-term durability. Post-curing may also influence composite density, void content, and filler–matrix interfacial integrity, which could indirectly affect the uniformity and reliability of radiation attenuation. Because post-curing was not evaluated in the present study, future work should systematically compare room-temperature curing with controlled thermal post-curing conditions and assess the resulting changes in density, tensile properties, FTIR spectra, thermal behavior, and Hp(10) attenuation performance under clinically relevant exposure conditions.

The computational analyses using the XCOM and Phy-X/PSD provided energy-dependent theoretical evaluation of the developed composites over a broad photon-energy range. XCOM allows calculation of photon cross sections and attenuation coefficients for elements, compounds, and mixtures over photon energies from 1 keV to 100 GeV, while Phy-X/PSD also supports photon-shielding and dosimetric analysis from 1 keV to 100 GeV. Therefore, the computational results provide sensitivity information across a wide energy domain, whereas the experimental validation was intentionally focused on Tc-99m because 140 keV photons are directly relevant to routine diagnostic nuclear medicine procedures and occupational exposure during Tc-99m handling. In the present study, both XCOM and Phy-X/PSD calculations showed that the attenuation response was strongly dependent on both photon energy and high-Z filler content. The incorporation of Bi_2_O_3_ and WO_3_ increased the MAC and LAC and reduced the HVL, TVL, and MFP relative to neat epoxy. These trends agree with numerous experimental and computational polymer studies in which greater high-Z filler loading increased composite density or effective atomic number and consequently reduced the material thickness required for attenuation [20,32,44]. The marked low-energy enhancement is consistent with the dominance of photoelectric absorption, whose probability is strongly influenced by the atomic number. At intermediate energies, differences between formulations decrease as Compton scattering becomes more important, while pair production contributes only above its threshold and is not relevant to Tc-99m photons at 140 keV. Additionally, the computational results are particularly consistent with prior XCOM, Monte Carlo, and Phy-X/PSD studies of Bi_2_O_3_- and WO_3_-containing polymers. Karabul and İçelli reported good agreement between experimental and MCNP6 results for Bi_2_O_3_/WO_3_ epoxy composites and found that nanoscale fillers attenuated photons more effectively than corresponding microscale fillers at equivalent concentrations [45]. Mahmoud et al. similarly predicted increasing LAC and decreasing HVL with increasing Bi_2_O_3_ concentration in the polyepoxide composites [46]. Studies using PMMA, LDPE, polypropylene, polyimide, silicone rubber, and polyester matrices have reported the same general loading-dependent behavior, demonstrating that the attenuation contribution of Bi_2_O_3_ and WO_3_ is reproducible across different polymer chemistries [19,20,47,48].

Correspondingly, experimental Hp(10) measurements confirmed the computational predictions under a clinically relevant Tc-99m exposure geometry. Increasing either Bi_2_O_3_ or WO_3_ loading progressively reduced the transmitted personal dose equivalent. At corresponding nominal loadings, nanoparticle-filled composites generally produced lower Hp(10) values than conventional-particle formulations. This result agrees with several studies reporting improved attenuation from nanoscale Bi_2_O_3_, WO_3_, SnO, and bismuth fillers because smaller particles can provide more extensive distribution of the attenuating phase and reduce particle-scale transmission pathways [18,43,49]. For example, Karabul and İçelli observed better attenuation from nano- Bi_2_O_3_ and nano- WO_3_ than from their microscale equivalents [45], while 3D-printed bismuth nanocomposites exhibited greater attenuation than bulk-particle formulations [50]. However, the particle-size effect is not universally monotonic: Hedaya et al. found that an optimized mixture of Bi_2_O_3_ micro- and nanoparticles outperformed formulations containing only one particle scale, indicating that particle packing and spatial distribution may be as important as nominal particle diameter [32]. Additionally, Bi_2_O_3_-filled composites generally attenuated Tc-99m photons more effectively than the corresponding WO_3_ formulations. At 140 keV, both fillers contribute through high-Z photoelectric interactions, but bismuth has a higher atomic number and Bi_2_O_3_ has a high intrinsic density, providing a strong attenuation contribution within the diagnostic nuclear-medicine energy range. This energy-dependent behavior is compatible with previous studies in which increasing Bi_2_O_3_ content improved attenuation at low photon energies, whereas tungsten-rich compositions could become advantageous at other energies depending on their absorption-edge positions, density, and composite architecture [20,43,47,48]. Nevertheless, experimental verification at additional medical photon energies, including approximately 50–150 keV for diagnostic X-ray and SPECT applications and 511 keV for PET annihilation photons, was not performed in the present study. In addition, build-up factors for different shielding thicknesses were not experimentally evaluated. Future studies should therefore include experimental validation across a wider medical photon-energy range and build-up factor assessment under broad-beam or scattered-radiation geometries to better support large-scale shielding design.

The most significant experimental finding was the superior shielding performance of the Bi-rich hybrid composite. The measured Hp(10) decreased progressively as the Bi_2_O_3_ proportion increased, with the hybrid 75:25 Bi_2_O_3_/WO_3_ NP formulation exhibiting the lowest transmitted personal dose equivalent among all investigated materials. Its mean Hp(10) value of 0.016 µSv was approximately 50% lower than that measured behind the lead reference under the same experimental geometry. This result supports a hybrid-filler design strategy in which WO_3_ contributes high density and photon attenuation, while the higher Bi_2_O_3_ fraction further enhances interaction with the 140 keV photons emitted by Tc-99m. Previous studies of Bi_2_O_3_/WO_3_-containing hybrid systems have likewise demonstrated composition-dependent attenuation, with the optimum filler ratio varying according to photon energy, filler loading, composite density, and material architecture [20,43,51,52].

Overall, the results demonstrate that hybrid Bi_2_O_3_/WO_3_ nanoparticle-filled epoxy composites are promising lead-free materials for Tc-99m radiation shielding. Structural and compositional analyses confirmed successful incorporation of the crystalline oxide fillers without substantial disruption of the cured epoxy network, while tensile testing indicated improved deformation capacity in the hybrid formulation. Both computational predictions and experimental Hp(10) measurements showed that radiation attenuation improved with increasing high-Z filler content. The hybrid 75:25 Bi_2_O_3_/WO_3_ NP composite exhibited the lowest Hp(10) and the best overall Tc-99m shielding performance. However, its comparison with lead is specific to the tested thickness and irradiation geometry. Based on the present findings, the developed Bi_2_O_3_/WO_3_ nanoparticle-filled epoxy composites are expected to exhibit reasonable radiation resistance under Tc-99m shielding conditions. This assumption is supported by the crosslinked thermoset structure of the cured epoxy matrix, the chemical stability of Bi_2_O_3_ and WO_3_ oxide fillers, and the absence of major FTIR spectral changes after filler incorporation, suggesting that the principal epoxy network was preserved. In addition, the experimental Hp(10) measurements confirmed effective attenuation under the investigated Tc-99m exposure geometry. Nevertheless, radiation resistance was not directly evaluated through long-term irradiation or repeated exposure-aging experiments in the present study. Therefore, future studies should evaluate density- and thickness-normalized attenuation, post-irradiation mechanical-property retention, FTIR spectral stability, surface morphology, filler leaching, thermal stability, and shielding efficiency after accumulated Tc-99m or gamma-ray exposure under clinically representative scattered-radiation conditions.

## 5. Conclusions

Bi_2_O_3_-, WO_3_-, and hybrid Bi_2_O_3_/WO_3_ nanoparticle-filled epoxy composites were successfully developed as lead-free polymer-based shielding materials for Tc-99m gamma radiation. Structural and chemical analyses confirmed effective filler incorporation without substantial disruption of the cured epoxy network, while mechanical testing showed that the hybrid formulation provided the greatest elongation at break. Both XCOM/Phy-X/PSD computational analyses and experimental Hp(10) measurements demonstrated improved attenuation with increasing high-Z filler content. The hybrid 75:25 Bi_2_O_3_/WO_3_ NP composite exhibited the lowest mean Hp(10) value of 0.016 µSv and achieved the best overall shielding performance among the tested formulations. These findings identify the Bi-rich hybrid epoxy composite as a promising candidate for further development as a customized lead-free radiation-protection material for nuclear medicine applications. Further validation should include density- and thickness-normalized attenuation measurements, post-curing optimization, long-term mechanical, thermal, and radiation stability, filler-leaching assessment, post-irradiation property retention, and testing under clinically representative scattered-radiation conditions.

## Figures and Tables

**Table 1:** Formulation and compositions of epoxy resin composites containing Bi_2_O_3_, WO_3_, and hybrid Bi_2_O_3_/WO_3_ nanoparticle fillers.

Sample	Epoxy Resin: Hardener Ratio	Filler System	Total Filler Loading * (phr)	Bi_2_O_3_:WO_3_ Ratio
Neat epoxy resin	3:1	None	0	—
25 Bi_2_O_3_	3:1	Bi_2_O_3_	25	100:0
50 Bi_2_O_3_	3:1	Bi_2_O_3_	50	100:0
75 Bi_2_O_3_	3:1	Bi_2_O_3_	75	100:0
100 Bi_2_O_3_	3:1	Bi_2_O_3_	100	100:0
25 WO_3_	3:1	WO_3_	25	0:100
50 WO_3_	3:1	WO_3_	50	0:100
75 WO_3_	3:1	WO_3_	75	0:100
100 WO_3_	3:1	WO_3_	100	0:100
Hybrid 25:75 Bi_2_O_3_/WO_3_	3:1	Bi_2_O_3_/WO_3_	100	25:75
Hybrid 50:50 Bi_2_O_3_/WO_3_	3:1	Bi_2_O_3_/WO_3_	100	50:50
Hybrid 75:25 Bi_2_O_3_/WO_3_	3:1	Bi_2_O_3_/WO_3_	100	75:25

* phr = 100 parts filler per 100 parts epoxy resin.

## Data Availability

The original contributions presented in this study are included in the article material. Further inquiries can be directed to the corresponding author.
